# Supplementary material for: Survival in a consecutive series of 467 glioblastoma patients: Association with prognostic factors and treatment at recurrence at two independent institutions
Source: PLoS One. 2023 Feb 2;18(2):e0281166. doi: 10.1371/journal.pone.0281166 (PMC9894455; doi:10.1371/journal.pone.0281166)
Supplement: S1 File — (DOCX) [file pone.0281166.s001.docx]

**Supplementary information**

**Methods**

**Data collection and eligibility for inclusion**

Demographic data including age and sex, and clinicopathological information including hemispheric tumour location (right, left and midline), anatomical tumour location (frontal, temporal, parietal, occipital, overlapping, and multifocal), tumour molecular genetic characteristics (*MGMT* promoter methylation and *IDH* mutation status), and treatment administered at primary diagnosis and at relapses were retrieved from patient records. Multifocality was registered if there were at least two distinct contrast-enhancing neoplastic foci independent of connecting T2/FLAIR signal abnormality. Surgical resection grade was determined based on examination of postoperative contrast-enhanced magnetic resonance imaging (MRI) in all cases and was classified as gross total resection (GTR, no residual contrast-enhancing tumour), subtotal resection (STR, residual contrast-enhancing tumour) or biopsy (1). In a few cases where minimal residual tumour or reactive changes were debated by the radiologist, the decision GTR/STR was also in conjunction with the neurosurgeon´s intraoperative assessment. Time-point of tumour progression was registered when MRI confirmed progression or recurrence based on increased contrast enhancement on T1 post-contrast sequence and/or increased pathological signal on T2/FLAIR weighted imaging which led to a change in treatment strategy, or death. Data were collected from medical records and survival data was obtained by reviewing death registers.

**Imaging and molecular pathology**

The MRI scanning protocol included 3D T1 pre-contrast, T2 axial, FLAIR axial and coronal, diffusion-weighted axial, and 3D T1 post-contrast sequences. Molecular pathological examinations were performed at the hospitals’ pathology departments following primary surgery. *MGMT* promoter methylation status was evaluated by polymerase chain reaction and verified by quantitative pyrosequencing. Mutations in the NADP-dependent isocitrate dehydrogenase genes (*IDH1* and *IDH2)* were investigated by Sanger sequencing or by immunohistochemistry for the *IDH1*^R132H^ oncometabolite 2-hydroxyglutarate.

**Results**

**Patient overall survival independent of treatment centre**

There was no significant difference in survival between patients treated at Haukeland University Hospital (HUH) and Oslo University Hospital (OUH), where patients treated at OUH (n=327) had 12.2 months compared to 11.8 months median overall survival of patients treated at HUH (n=140); HR_1.14_, 95% CI [0.93-1.41], *p*=0.201, Fig 1B. Overall, 21.4% of patients were alive at 2 years, 95% CI [0.18-0.25] and 6.8%, 95% CI [0.04-0.11] were alive at 5 years.

**Treatment options upon tumour recurrence differed between the two institutions**

At first tumour recurrence, the major difference in treatment practice was that 43.3% (91/210) of patients were administered chemotherapy at OUH. In contrast, HUH more frequently administered GK/SRS to 30.3% (30/99) followed by LAVA to 18.2% (18/99) of patients at first recurrence. The fraction of patients not receiving further treatment was similar at OUH and HUH, 32.9% *vs.* 27.3%, respectively (Table 3).

At second recurrence, patients were more frequently administered conventional chemotherapy at 24.2% (22/91) at OUH *vs.* 8.2% (5/61) at HUH. The latter more frequently administered LAVA to 32.8% (20/61) and GK/SRS alone or combined with chemotherapy to 16.4% (10/61) of patients at second recurrence. Median time to first progression was 8.6 *vs.* 6.6 months for OUH and HUH, respectively.

Approximately 64.3% (36/56) of patients from HUH were alive to be diagnosed with a third tumour recurrence; however, 47.2% (17/36) of these patients were not administered further antineoplastic treatment. Approximately 38.9% (14/36) received LAVA treatment, 5.6% (2/36) received GK/SRS and 8.3% (3/36) received re-operation alone or combined with chemotherapy at the third recurrence. In contrast, 35.7% (20/56) of patients from OUH achieved a third MRI-confirmed recurrence and the majority 80% (16/20) did not get further treatment. The handful of patients 20% (4/20), who were eligible for further antineoplastic treatment received either re-irradiation or chemotherapy alone or in combination with bevacizumab. Only patients treated with LAVA were alive 12 months after their third recurrence (7.1%).

**Characteristics of longest surviving patients**

Amongst the patients with tumours harbouring hypermethylated *MGMT* promoter, 38.8%, 95% CI [0.32-0.46] and 12.8%, 95% CI [0.06-0.21] were alive at 2 and 5 years, respectively, compared to 9.8%, 95% CI [0.06-0.14] with unmethylated promoter at 2 years. No patients harbouring unmethylated *MGMT* promoter were alive at 5 years, Table 1. Amongst patients who were younger than 60 years 31.6%, 95% CI [0.250-0.38] and 11.6%, 95% CI [0.06-0.20] were alive after 2 and 5 years respectively, compared to 22.9%, 95% CI [0.16-0.30] and 4.6 %, 95% CI [0.02-0.10] of those older (60-69 years). Only 5.8 %, 95% CI [0.03-0.11] of patients 70 years and older were alive after 2 years and 3.9%, 95% CI [0.01-0.10] were alive after 5 years. Of patients with wild type *IDH* only 5.5%, 95% CI [0.03-0.10]) were alive after 5 years, in contrast to 12.2%, 95% CI [0.01-0.37] of patients with mutated *IDH* gene. More patients with tumour location in the left hemisphere (14.6%, 95% CI [0.09-0.21]) were alive after 5 years compared to 2.1%, 95% CI [0.005-0.06] with tumour location in the right hemisphere, and patients with midline or bilateral tumour location 4.6%, 95% CI [0.01-0.12]. Those who had their tumour gross totally resected (11.6%, 95% CI [0.06-0.20]) were alive after 5 years compared to 6.2%, 95% CI [0.03-0.11] of patients who received subtotal tumour resection.

Only patients who received 60 Gy IR with concomitant TMZ (9.3%, 95% CI [0.05-0.15]) or hypofractionated <60 Gy with concomitant TMZ (2.8%, 95% CI [0.003-0.11]) at primary diagnosis were among patients surviving 5 years. No treatment administered at recurrence was associated with survival benefit at 5 years from the start of recurrent treatment, however, 11.5% of patients who received gamma knife/SRS or re-operation alone or in combination with chemotherapy at first recurrence were alive 2 years after first recurrence. Patients who received LAVA or conventional chemotherapy (9.1 *vs.* 8 %, respectively), were alive at 2 years after first recurrence.

**References**

1. Oszvald A, Güresir E, Setzer M, Vatter H, Senft C, Seifert V, Franz K: Glioblastoma therapy in the elderly and the importance of the extent of resection regardless of age. J Neurosurg 116:357-364, 2012.

**Supplementary figure legend**

**S1 Fig.** **Effect of treatment administered at subsequent tumour recurrence on patients’ overall survival**

(A) Cumulative overall (%) survival time in months and 95% confidence intervals after 1 year, Cumulative overall (%) survival time from diagnosis in months after 1-, 2 -and 5-year follow-up for (B) treatment administered after first tumour recurrence, (C) treatment administered after second tumour recurrence, and (D) treatment administered after third tumour recurrence. LAVA: lomustine, bevacizumab, and vincristine; TMZ: temozolomide; IR: ionizing radiation; Gy: gray; SRS: stereotactic radiosurgery; GK: gamma knife; Other: GK/SRS/IR (+/- chemotherapy), Chemotherapy, Surgery (+/- chemotherapy).
