# Supplementary figures and images for: Survival in a consecutive series of 467 glioblastoma patients: Association with prognostic factors and treatment at recurrence at two independent institutions
Source: PLoS One. 2023 Feb 2;18(2):e0281166. doi: 10.1371/journal.pone.0281166 (PMC9894455; doi:10.1371/journal.pone.0281166)

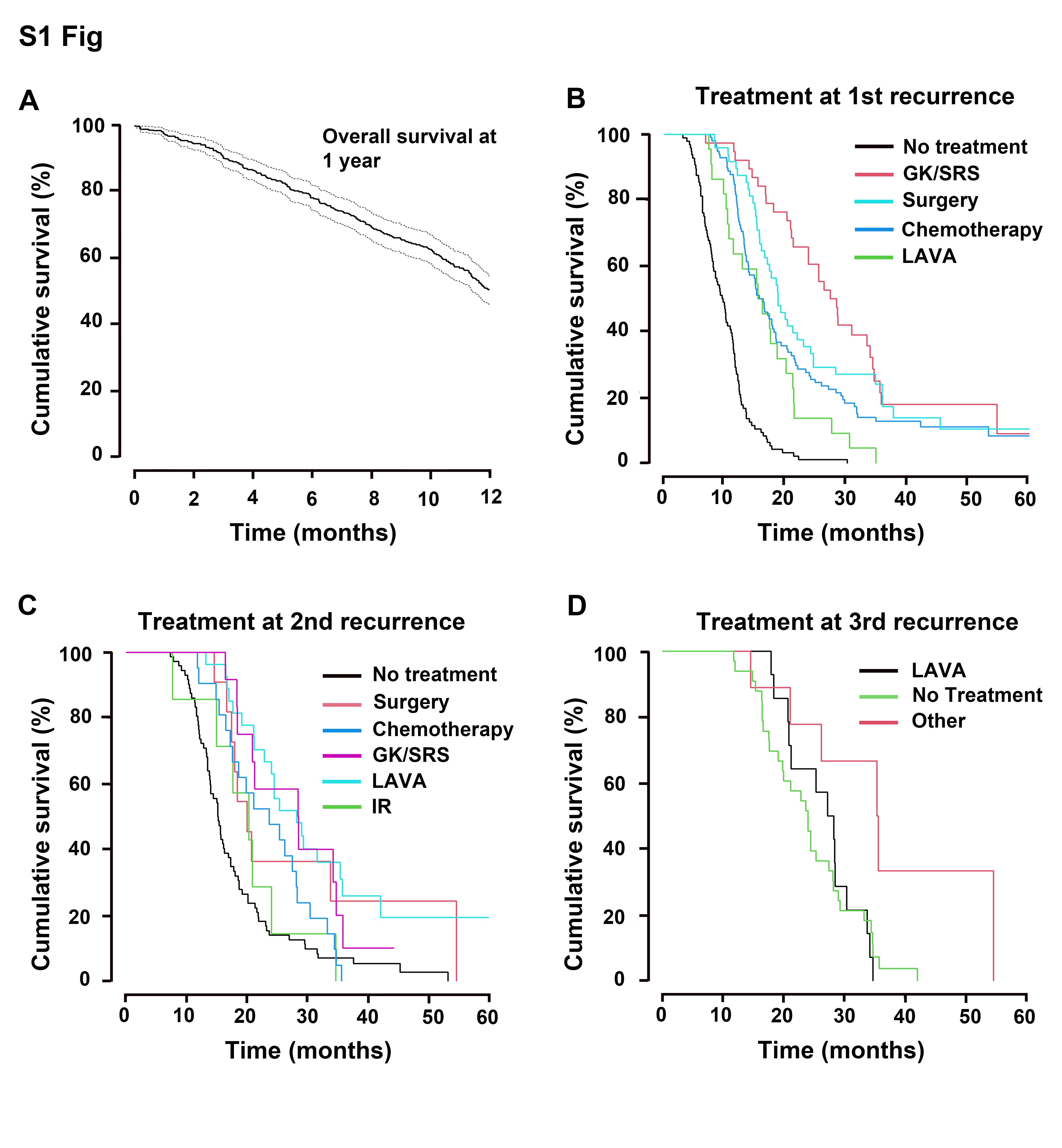

Supplement: S1 Fig — (A) Cumulative overall (%) survival time in months and 95% confidence intervals after 1 year, Cumulative overall (%) survival time from diagnosis in months after 1-, 2 -and 5-year follow-up for (B) treatment administered after first tumour recurrence, (C) treatment administered after second tumour recurrence, and (D) treatment administered after third tumour recurrence. LAVA: lomustine, bevacizumab and vincristine; TMZ: temozolomide; IR: ionizing radiation; Gy: gray; SRS: stereotactic radiosurgery; GK: gamma knife; Other: GK/SRS/IR (+/- chemotherapy), Chemotherapy, Surgery (+/- chemotherapy). (TIF) [file pone.0281166.s002.tif]
