# Supplementary material for: Survival in a consecutive series of 467 glioblastoma patients: Association with prognostic factors and treatment at recurrence at two independent institutions
Source: PLoS One. 2023 Feb 2;18(2):e0281166. doi: 10.1371/journal.pone.0281166 (PMC9894455; doi:10.1371/journal.pone.0281166)
Supplement: Attachment — Submitted filename: Rebuttal PLoS one -PONE-D-22-23881[47].docx [file pone.0281166.s003.docx]

**1. Please ensure that your manuscript meets PLOS ONE's style requirements, including those for file naming. The PLOS ONE style templates can be found at**

<https://journals.plos.org/plosone/s/file?id=wjVg/PLOSOne_formatting_sample_main_body.pdf> and

<https://journals.plos.org/plosone/s/file?id=ba62/PLOSOne_formatting_sample_title_authors_affiliations.pdf>

*Authors: Thank you. We have formatted the manuscript as requested.*

**2. We note that the grant information you provided in the ‘Funding Information’ and ‘Financial Disclosure’ sections do not match. When you resubmit, please ensure that you provide the correct grant numbers for the awards you received for your study in the ‘Funding Information’ section.**

*Authors: Thank you. The information provided in the “funding information” section is correct.*

**3. Thank you for stating the following in the Acknowledgments Section of your manuscript:**

**"We are grateful to the Norwegian Cancer Society (grant # 190170) and KLINBEFORSK for supporting our research. "**

**We note that you have provided funding information that is not currently declared in your Funding Statement. However, funding information should not appear in the Acknowledgments section or other areas of your manuscript. We will only publish funding information present in the Funding Statement section of the online submission form.**

**Please remove any funding-related text from the manuscript and let us know how you would like to update your Funding Statement. Currently, your Funding Statement reads as follows:**

**"1. MC**

**Grant # 190170**

**Norwegian Cancer Society**

[**https://kreftforeningen.no/**](https://kreftforeningen.no/)

**Sponsors or funders did not play any role in the study design, data collection and analysis, decision to publish, or preparation of the manuscript**

**2. MC and DG**

**Program for klinisk behandlingsforskning - KLINBEFORSK**

[**https://kliniskforskning.rhf-forsk.org/**](https://kliniskforskning.rhf-forsk.org/)

**Sponsors or funders did not play any role in the study design, data collection and analysis, decision to publish, or preparation of the manuscript"**

**Please include your amended statements within your cover letter; we will change the online submission form on your behalf.**

*Authors: we have now removed the acknowledgement of funding from the manuscript. We confirm that the information provided in the “funding information” section is correct.*

**4. We note that you have indicated that data from this study are available upon request. PLOS only allows data to be available upon request if there are legal or ethical restrictions on sharing data publicly. For more information on unacceptable data access restrictions, please see** [**http://journals.plos.org/plosone/s/data-availability#loc-unacceptable-data-access-restrictions**](http://journals.plos.org/plosone/s/data-availability#loc-unacceptable-data-access-restrictions)**.**

**In your revised cover letter, please address the following prompts:**

**a) If there are ethical or legal restrictions on sharing a de-identified data set, please explain them in detail (e.g., data contain potentially sensitive information, data are owned by a third-party organization, etc.) and who has imposed them (e.g., an ethics committee). Please also provide contact information for a data access committee, ethics committee, or other institutional body to which data requests may be sent.**

**b) If there are no restrictions, please upload the minimal anonymized data set necessary to replicate your study findings as either Supporting Information files or to a stable, public repository and provide us with the relevant URLs, DOIs, or accession numbers. For a list of acceptable repositories, please see** <http://journals.plos.org/plosone/s/data-availability#loc-recommended-repositories>.

*Authors: Data cannot be shared publicly because of this is sensitive patient data. Due to these restrictions on public sharing for participant privacy, Data approved by the Ethics Committee (approval number 2017/2084) will be available from the corresponding authors upon request.*

**We will update your Data Availability statement on your behalf to reflect the information you provide.Please include your full ethics statement in the ‘Methods’ section of your manuscript file. In your statement, please include the full name of the IRB or ethics committee who approved or waived your study, as well as whether or not you obtained informed written or verbal consent. If consent was waived for your study, please include this information in your statement as well.**

*Authors: This information that was previously in the supplementary information, is now added to the main manuscript.*

*Full name of the IRB or ethics committee and contact details:*

***Regional Ethics Committee, REK vest***

*Armauer Hansens House (AHH),*

*Tverrfløy Nord, 2 floor. Room*

*281. Haukelandsveien 28, 5025 Bergen, Norway*

*Telefon: 55975000*

*E-post: rek-vest@uib.no*

*Web: http://helseforskning.etikkom.no/*

*Kindly address all mail and e-mails to the Regional Ethics Committee, REK vest, not to individual staff*

***Non-author Contact person:***

*Berit Bølge Tysnes, PhD*

*Department of Biomedicine*

*University of Bergen*

*Jonas Lies vei 91*

*5009 Bergen, Norway*

*Email: Berit.Tysnes@uib.no*

*Phone: +4790778791 / +4755586093*

**5. Please include your full ethics statement in the ‘Methods’ section of your manuscript file. In your statement, please include the full name of the IRB or ethics committee who approved or waived your study, as well as whether or not you obtained informed written or verbal consent. If consent was waived for your study, please include this information in your statement as well.**

*Authors:* Regional Committee for Medical and Research Ethics for Western Norway (REC West) approved the retrospective patient identification and collection of clinicopathological data (2017/2084). Exemption from the need to obtain informed consent from included patients, including the few surviving patients at the time of data collection (*n*=9 and *n*=43; HUH and OUH, respectively), was granted by REC West.

**6. Please review your reference list to ensure that it is complete and correct. If you have cited papers that have been retracted, please include the rationale for doing so in the manuscript text, or remove these references and replace them with relevant current references. Any changes to the reference list should be mentioned in the rebuttal letter that accompanies your revised manuscript. If you need to cite a retracted article, indicate the article’s retracted status in the References list and also include a citation and full reference for the retraction notice.**

*Authors:* References have been reviewed and corrected.

Additional Editor Comments:

**Kindly respond to the reviewers comments, especially regarding treatment strategy at initial occurrence and at recurrence, and the risk of bias.**

**Authors:** To analyse the impact of the previous treatment on the reccurent treatments, analysis using time-dependent covariates were performed. Furthermore, to avoid time immortal bias, we set a landmark conditioning on patients reaching 1^st^, 2^nd^ and 3^rd^ recurrence.

Reviewers' comments:

**Comments to the Author**

5. Review Comments to the Author

Please use the space provided to explain your answers to the questions above. You may also include additional comments for the author, including concerns about dual publication, research ethics, or publication ethics. (Please upload your review as an attachment if it exceeds 20,000 characters)

Reviewer #1: The reviewed manuscript Survival in a consecutive series of 467 glioblastoma patients: association with prognostic factors and treatment at recurrence at two independent institutions is well structured and neatly presented retrospective study that sheds light on outcomes in treatment of recurrent glioblastoma, considering the intrinsic biological, pathological and clinical features of the disease, as well as primary therapeutic modalities.

The manuscript is delivered in a clear and precise, easily comprehensible manner. The methodology is sufficiently detailed, the results are presented in illustrative tables and figures, and conclusions are adequate and well elaborated.

The problem analyzed in the present study is of major clinical relevance. There is no established treatment for GBM recurrence, thus the results of the present study may be significant in treatment decision making for patients and may contribute to the improvement of patients' outcomes. Given that the research includes two centers with, to some extent, different treatment approaches, the results of the study may represent indicative guidepost for the treatment of recurrent GBM depending on the available resources at the institution.

It was a pleasure to read this manuscript. I find this article acceptable to be published in present form.

*Authors: We are grateful to the reviewer for constructive feedback that greatly improved the quality of the work.*

Reviewer #2: Thank you for the opportunity to review your work. The authors investigated physicians’ treatment choice at recurrence and prognostic and predictive factors for survival in GBM patients from Norway’s two largest regional hospitals.

1) **there are a few grammatical errors here and there that may require a review**

Authors: Thank you, we have now thoroughly proof read the manuscript and corrected all grammatical errors.

2) **the prognosis factors associated with GBM has been investigated in published studies, no additional information could be available from present study.**

Authors: This is indeed true. We agree with the reviewer. We controlled for these established biomarkers in our study in order to provide quality assurance of our patient cohort, ensuring that the findings are as expected from the literature.

**3) The treatment strategy at second recurrence would be significantly impacted by first-line treatment. For example, 50% of GBM recurred in one year, if GBM received first-line concurrent chemotherapy, most radiation oncologists would recommend to other treatment but not for radiotherapy due to its high-risk of toxicities. As a result, the analysis of treatment strategy at recurrence is obviously biased in the present study.**

*Authors:* *We agree with the reviewer that the treatment at the second recurrence is impacted by the first line treatment. We did attempt to control the inherent bias with such retrospective analyses. To analyse the impact of the previous treatment on the recurrent treatments, analysis using time-dependent covariates were performed. Furthermore, to avoid time immortal bias, we set a landmark conditioning on patients reaching 1^st^, 2^nd^ and 3^rd^ recurrence. We totally agree that the re-irradiation is not recommended during the first year following postoperative radiotherapy. However, those limitations do not apply to the stereotactic radiosurgery/GK, which due to the limited volume of irradiated tissue and little toxicity, is often the treatment of choice for small contrast enhancing relapses. The selection of chemotherapy regimen at relapse/progression also depends on timing of treatment failure/relapse. Progressive disease during adjuvant therapy precludes use of temozolomide, indicating the primary resistance. We have now included this statement in the discussion.*

*Those real-life data elucidating the available treatment options may be useful for clinicians to select treatment for recurrent glioblastoma patients in the paucity of positive clinical trials. We propose that, however limited, the available therapies may be considered for individual approach.*
